# Sestrin2 Exerts a Novel Protective Effect Against LPS‐Induced Ferroptosis via the Nrf2–SLC7A11–GPX4 Signaling Axis

**DOI:** 10.1096/fj.202501348RRR

**Published:** 2025-11-26

**Authors:** Xuerui Zhang, Jiawei Yin, Yuan Yang, Jie Peng, Yu Xu, Wenting Zhang, Haodong Xiao, Zupeng Lu, Huanyu Liu, Yanjun Wen, Miaomiao Liu, Enguang Chen, Xiang Zhang, Shipeng Guo, Lianhui Han, Huazhang Feng, Peiquan Zhao

**Affiliations:** ^1^ Department of Ophthalmology Xinhua Hospital Affiliated to Shanghai Jiao Tong University School of Medicine Shanghai China; ^2^ Shanghai Jiao Tong University School of Medicine Shanghai China; ^3^ Department of Ophthalmology, Shanghai Children's Hospital, School of Medicine Shanghai Jiao Tong University Shanghai China; ^4^ Department of Ophthalmology, Eye & ENT Hospital, Shanghai Medical College Fudan University Shanghai China; ^5^ Changxing People's Hospital of Chongming District Shanghai China

**Keywords:** ferroptosis, inflammation, LPS‐induced uveitis, Nrf2, SESN2

## Abstract

Uveitis is a leading cause of visual impairment linked to systemic inflammatory diseases. Current corticosteroid treatments are limited by significant long‐term side effects. Sestrin 2 (SESN2), a stress‐responsive antioxidant protein, is implicated in the regulation of ferroptosis and inflammation. This study investigates SESN2's role in modulating inflammation and ferroptosis in endotoxin‐induced uveitis (EIU) models, aiming to develop safer therapeutic alternatives for uveitis management. C57BL/6 mice were subjected to EIU via intravitreal injection of lipopolysaccharide (LPS) with or without SESN2‐overexpressing adeno‐associated virus (AAV). Clinical evaluations, cytokine profiling, and ferroptosis marker assessments were conducted. Additionally, BV2 microglial cells were genetically modified to overexpress or silence SESN2, followed by analyses of inflammatory cytokine production, oxidative stress, ferroptosis‐related pathways, mitochondrial function, and involvement of the Nrf2 signaling pathway. SESN2 expression was significantly reduced in vivo and in vitro following LPS treatment. AAV‐mediated SESN2 overexpression suppressed inflammatory responses. Mechanistically, SESN2 inhibited ferroptosis by upregulating ferroptosis‐resistant proteins (SLC7A11, GPX4) and reducing lipid peroxidation markers (4‐HNE). In vitro, SESN2 overexpression reduced oxidative stress and ferroptosis, while SESN2 knockdown exacerbated these effects. SESN2's protective role was mediated by Nrf2 activation, enhancing antioxidant defenses and ferroptosis inhibition. Inhibition of Nrf2 reversed SESN2's protective effects, underscoring the importance of the p62/Nrf2/GPX4 axis in SESN2‐mediated ferroptosis protection. SESN2 plays a crucial role in protecting against inflammation and ferroptosis via the Nrf2 pathway, presenting a promising therapeutic target for uveitis management.

## Graphical abstract

This graphical abstract shows that LPS downregulates SESN2, leading to suppressed GPX4 activity, increased ROS, lipid peroxidation, and inflammation. SESN2 overexpression reverses these effects by activating the Nrf2/SLC7A11/GPX4 axis, reducing MDA and 4HNE levels, preserving redox balance, and alleviating ocular symptoms including hypopyon and retinal vascular inflammation.

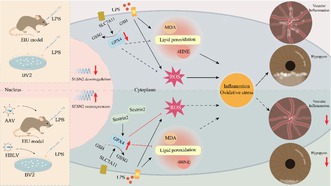

**Abbreviations**
4HNE: 4‐hydroxynonenal
FTH1: ferritin heavy chain 1
GPX4: glutathione peroxidase 4
GSH: glutathione
GSSG: glutathione disulfide
MDA: malondialdehyde
Nrf2: nuclear factor‐erythroid 2‐related factor 2
SESN2: Sestrin 2
SLC7A11: solute carrier family 7 member 11
SOD1/2: superoxide dismutase 1/2

## 1 Background

Uveitis is a critical component of multisystem inflammatory diseases, serving as a key indicator of underlying autoimmune disorders, infections, or systemic chemical exposures [1, 2]. This group of intraocular inflammatory conditions affects the uveal tract, making uveitis a leading cause of severe visual impairment and contributing to 10%–20% of legal blindness in developed countries [3, 4, 5]. Clinically, uveitis presents with symptoms such as redness, photophobia, and blurred vision, and can progress to blindness due to chronic and recurrent inflammation that damages ocular tissues [6, 7]. Although corticosteroids remain the standard treatment for suppressing inflammation, their long‐term use poses significant risks, including increased intraocular pressure, cataracts, and glaucoma [8]. Consequently, the development of safer and more sustainable therapeutic alternatives is imperative for effectively managing this complex, multisystem‐associated condition.

Endotoxin‐induced uveitis (EIU), a well‐established animal model of uveitis triggered by lipopolysaccharide (LPS), is characterized by the infiltration of inflammatory cells into the anterior segment and blood‐aqueous barrier disruption. The acute inflammatory response typically begins approximately 4 h following LPS injection, reaches its peak between 18 and 24 h, and typically resolves within 48 h [9, 10]. EIU has been widely used to investigate the pharmacological and immunological effects of therapeutic agents on intraocular inflammation.

Ferroptosis, a programmed cell death characterized by lipid peroxidation induced by reactive oxygen species (ROS), is iron‐dependent and contributes to various pathological conditions, including degenerative diseases, cancer, and inflammatory disorders [11]. Studies of inflammation‐related intestinal diseases, such as colitis and colitis‐associated cancer, have identified ferroptosis as a crucial mediator that amplifies inflammatory responses through the release of danger‐associated molecular patterns [12]. Accumulating evidence indicates that pharmacological inhibition of ferroptosis effectively mitigates LPS‐induced inflammatory responses, as demonstrated by reduced tissue injury and decreased proinflammatory cytokine production [13, 14, 15]. Furthermore, ferroptosis demonstrates a significant role in uveitis pathogenesis, particularly in conditions exacerbated by environmental factors such as PM2.5 exposure [16, 17]. Therefore, it is postulated that ferroptosis may contribute to the onset, progression, and potentially the prognosis of uveitis.

Sestrin 2 (SESN2), a highly conserved stress‐responsive protein, is activated by various cellular stressors including oxidative stress, DNA damage, and hypoxia [18]. This protein demonstrates crucial functions in alleviating endoplasmic reticulum stress, promoting autophagy, and reducing apoptosis [19, 20]. As an efficient antioxidant, SESN2 facilitates ROS elimination and exhibits protective effects in various ocular conditions, including glaucoma and cataracts [21, 22, 23, 24]. Recent studies have highlighted SESN2's protective role in cardiomyocytes during LPS‐induced septic cardiac injury and its regulation of ferroptosis in dendritic cells [25, 26]. While SESN2 is critical in maintaining cellular homeostasis under stress conditions, its potential involvement in iron overload‐induced uveitis remains unexplored. Given SESN2's antioxidant properties and its influence on ferroptosis, we hypothesize that SESN2 may modulate ferroptosis in LPS‐induced inflammation. This study aims to investigate SESN2's regulatory effects on ferroptosis in LPS‐induced inflammation and elucidate the underlying signaling pathways in uveitis.

## 2 Methods

### 2.1 EIU Mouse Model and Interventions

Female C57BL/6 mice (6–8 weeks old, 18–20 g, RRID:IMSR_JAX:000664) were purchased from the Shanghai Jihui Laboratory Animal Care Company. The animals were maintained in a controlled environment (temperature: 20°C–24°C; 12 h light/dark cycle) with unrestricted access to food and water. All experimental protocols were approved by the Institutional Animal Care and Use Committee of Xinhua Hospital, affiliated with the School of Medicine at Shanghai Jiao Tong University (Approval No. XHEC‐NSFC‐2023‐145) and performed in compliance with the guidelines established by the National Institutes of Health (NIH) for laboratory animals.

For the induction of acute uveitis, EIU was initiated via a single intravitreal injection of 1 μL of lipopolysaccharide (LPS, 125 ng/μL; Sigma‐Aldrich). Experimental mice were randomly assigned to receive either a vehicle (phosphate‐buffered saline, PBS) or adeno‐associated virus (AAV) via intravitreal injection, three weeks prior to LPS treatment. Intraocular inflammation associated with EIU develops acutely, with clinical manifestations appearing at 24 h post‐injection, and resolving by 48 h post‐treatment [27]. Retinas were collected 24 h after LPS injection. Animals with injection‐related trauma, lens injury, media opacity, or no inflammatory response were excluded. All animals that developed typical signs of anterior uveitis (such as iris vessel dilation and aqueous flare) within 24 h after LPS injection were included. They were randomly assigned to one of four experimental groups: (1) control group (PBS), (2) LPS‐stimulated group (LPS), (3) LPS with control AAV group (LPS+AAV‐CON), and (4) LPS with SESN2‐overexpressing AAV (LPS+AAV‐SESN2). In the LPS+AAV groups, a single intravitreal injection of 1 μL AAV‐CON or AAV‐SESN2 was administered. Sample size was determined based on a power analysis with a significance level of 0.05 and a power of 0.8. All outcome assessments and data analyses were performed by investigators blinded to group allocation.

### 2.2 AAV Preparation

The murine *Sesn2* open reading frame (ORF) was amplified with specific primers (forward: 5′‐CCATAGAAGACACCGGCTAGCGGATCCGCCACCATGATCGTAGCGGACT‐3′; reverse: 5′‐TCATCGTCATCCTTGTAGTCGGTACCGGTCATGTAGCGGGTGATGGC‐3′) and inserted into an entry plasmid to generate a recombinant AAV construct expressing *Sesn2*. Recombinant AAV particles were generated using the pHBAAV‐CMV‐MCS‐T2A‐mcherry gateway plasmid, as per the manufacturer's instructions (Hanbio Biotechnology). The viral particles (1 × 10^9^ pfu) were dissolved in a 0.001% Pluronic F‐68 formulation buffer and administered intraocularly. DNA sequence verification of the recombinant AAV was conducted using Phanta Super‐Fidelity DNA Polymerase. For AAV packaging, the *Sesn2* construct was co‐transfected into AAV‐293 cells with pAAV‐RC, pHelper, and shuttle vectors using the Lipofiter transfection reagent (Hanbio Biotechnology). After 72 h, cells were harvested, and viral particles were purified using CsCl2 density gradient centrifugation. The viral preparation was treated with Benonase to remove residual plasmid and genomic DNA, concentrated via ultrafiltration, and stored at −80°C. Viral titers were determined by quantitative real‐time PCR (qPCR) using primers specific to the ITR and WPRE sequences, yielding a final titer of 1.3 × 10^12^ vg/mL. AAV expressing mCherry was used as the infection control.

### 2.3 Flat‐Mount Immunofluorescence Staining

Eyeballs were collected and fixed in 4% paraformaldehyde for 45 min after a small corneal incision. The anterior segment was removed to isolate the retina. Retinas were blocked in antibody diluent and incubated overnight at 4°C with anti‐IBA1 antibody (Fujifilm Wako, Cat# 019‐19741, RRID:AB_839504). The following day, secondary antibody (Invitrogen, A‐11070; RRID:AB_2534114) was applied for 2 h at room temperature. Retinas were then radially incised into four quadrants, flat‐mounted using antifade mounting medium with DAPI (Meilunbio, Cat# MA0222), and imaged with a fluorescence microscope.

### 2.4 Cell Culture and Genetic Manipulation

The microglial cell line obtained from the AoYin Cell Center cultured in DMEM was verified to be mycoplasma‐free (10% FBS, 1% penicillin–streptomycin) (Thermo Fisher Scientific). Cells were maintained until approximately 80% confluence and serum‐starved for 12 h prior to treatment. To activate cells, 200 ng/mL LPS was added in serum‐free media, while controls received serum‐free media alone. After 24 h of incubation at 37°C with 5% CO_2_, cells were collected. Media were removed, and cells were washed twice with PBS (Servicebio Technology), then stored at −80°C. All experiments were performed in triplicate and repeated with different passage numbers.

For SESN2 silencing, BV2 cells were transfected with *Sesn2*‐targeted siRNA (si‐SE, GenePharma) using Lipofectamine 3000 (Thermo Fisher Scientific). siRNA sequences are listed in Table 1. A final concentration of 50 nM si‐*Sesn2* was used, and cells were incubated with 200 ng/mL LPS for either 24 or 48 h, depending on the assay. A scrambled siRNA (si‐CON) was used as a control.

**TABLE 1:** siRNA sequences.

siRNA	Sense siRNA sequence (5′‐3′)	Antisense siRNA sequence (5′‐3′)
si‐SE	GCGAGGUCAACAAGUUACU tt	AGUAACUUGUUGACCUCGC tt

*Note:* SESN2 siRNA sequences. The table lists the sense and antisense siRNA sequences (5′‐3′) designed to target SESN2 (si‐SE).

Lentivirus vectors encoding the mouse *Sesn2* gene were inserted into the PGMLV‐CMV‐MCS‐3×Flag‐PGK‐Puro plasmid. BV2 cells were transduced with lentivirus after reaching 80% confluence in six‐well plates. Lentivirus was added to polybrene‐supplemented media, and cells were incubated for 48 h. Viral packaging and purification were conducted by co‐transfecting HEK293T cells (RRID:CVCL_0063) with packaging vectors. BV2 cells were then transduced with *Sesn2*‐overexpressing lentivirus for experimental analysis. Six experimental groups were established: control (PBS), LPS‐stimulated (LPS), LPS with control lentivirus (LPS+HBLV‐CON), LPS with *Sesn2*‐overexpressing lentivirus (LPS+HBLV‐SE), LPS with control siRNA (LPS+si‐CON), and LPS with *Sesn2*‐silencing siRNA (LPS+si‐SE).

### 2.5 qPCR

Total RNA was extracted from cultured BV2 cells and mouse retinal tissues using the EZ‐press RNA Purification Kit (EZBioscience). RNA concentration was determined with a microplate spectrophotometer. One microgram of RNA was reverse‐transcribed into cDNA using the HiScript II Q RT SuperMix (Vazyme) according to the manufacturer's instructions. Quantitative real‐time PCR was performed using the ChamQ Blue Universal SYBR qPCR Master Mix (Vazyme) on a real‐time PCR system. Each reaction was run in triplicate. β‐actin served as the internal reference gene, and relative gene expression was calculated using the 2^−ΔΔCT^ method. Primer sequences are listed in Table S1.

### 2.6 Western Blotting (WB)

Protein extracts were obtained from BV2 cells or retinal tissues by lysis with RIPA buffer (Meilunbio Biotechnology), containing protease inhibitors (Abclonal Technology) and a phosphatase inhibitor cocktail (Meilunbio Biotechnology). The samples were sonicated for 10 s and centrifuged (12 000 *g*, 10 min, 4°C) to remove debris. Protein concentrations were quantified using the bicinchoninic acid (BCA) assay (GBCBIO Technologies). Following the transfer of separated proteins onto PVDF membranes (Millipore), the membranes were incubated with TBST containing either 5% skim milk or 1% BSA for blocking. The membranes were incubated with primary antibodies including GADPH (Proteintech Cat# CL488‐60004, RRID:AB_2919223), p62 (Abcam Cat# ab109012, RRID:AB_2810880), SLC7A11 (Proteintech Cat# 26864‐1‐AP, RRID:AB_2880661), FTH1 (4393T; Cell Signaling Technology, RRID:AB_11217441), SOD1 (Affinity Biosciences Cat# AF5198, RRID:AB_2837684), SOD2 (Proteintech Cat# 24127‐1‐AP, RRID:AB_2879437), GPX4 (Proteintech Cat# 30388‐1‐AP, RRID:AB_3086304), 4‐HNE (Bioss Cat# bs‐6313R, RRID:AB_2827741), NRF2 (Proteintech Cat# 80593‐1‐RR, RRID:AB_2918904), P‐NRF2 (Bioss Cat# BSM‐52179R, RRID:AB_3492114), and SESN2 (Proteintech Cat# 66297‐1‐Ig, RRID:AB_2881680). Quantitative analysis was conducted using ImageJ software (RRID:SCR_003070).

### 2.7 Enzyme‐Linked Immunosorbent Assay (ELISA)

BV2 cells were harvested for cytokine analysis after reaching 70%–80% confluence in six‐well plates. Cells were transduced with lentiviral vectors for *Sesn2* overexpression or transfected with siRNA for *Sesn2* silencing. Conditioned media were collected, and cytokine levels (IL‐15, IL‐1β, ICAM‐1, TNF‐α) were quantified using ELISA kits, in accordance with the manufacturer's protocol (Meimian Biotechnology).

### 2.8 Clinical Evaluation and Electroretinogram (ERG)

At 24 h post‐LPS administration, mice were anesthetized via intraperitoneal injection of sodium pentobarbital (50 mg/kg) and administered propantheline and tropicamide. The clinical assessment of EIU was conducted through the evaluation of iris blood vessel dilation, fibrinous leakage, hypopyon, and pupil occlusion. To enhance objectivity, clinical scoring was performed by three independent observers who were blinded to the treatment groups. The scoring system, detailed in Table S2, follows a widely accepted semi‐quantitative method extensively used in the EIU model [28, 29, 30, 31]. Inflammatory responses in the anterior segment were assessed using a slit lamp biomicroscope and the fundus imaging system (Optomap 200Tx). Ofloxacin ointment was applied post‐examination to protect the cornea.

ERG recordings were performed after 8–12 h of dark adaptation to assess retinal function. Mice were anesthetized with pentobarbital sodium, and pupils were dilated with 1% mydriacyl. 3% Hypromellose gel was applied for lubrication, and additional anesthesia was administered with 0.5% tetracaine eye drops. ERG signals were recorded using gold‐plated wire loop electrodes on the corneal surface, with steel needles positioned near the middle of the forehead and tail acting as reference and ground electrodes. Flash stimuli were applied at different intensities (0.1, 3.0 cd s/m^2^). a‐wave and b‐wave amplitudes were averaged from three responses per stimulus intensity and analyzed using the Espion Diagnosys System (Diagnosys LLC).

### 2.9 Histopathological Analysis

Mouse eyeball tissues were fixed in 4% PFA and then embedded in paraffin. Cross‐sections (5 μm) of the optic nerve were stained with hematoxylin and eosin (H&E) [32]. The optic disc sections were visualized using an Olympus BX51 microscope.

### 2.10 Intracellular Iron Detection

Intracellular Fe^2+^ levels were quantified by a FerroOrange Kit (Dojindo) following the provided instructions. Cells were incubated with FerroOrange in 5% CO_2_ (1 μmol/L, 60 min, 37°C). Fluorescence images were acquired using a Nikon Fi3 fluorescence microscope, with random fields selected. Intensity was quantified using ImageJ software (RRID:SCR_003070) and normalized to the control group.

### 2.11 Transmission Electron Microscopy (TEM) Observation

BV2 cells were harvested from six‐well plates, pelleted by centrifugation, and fixed with 2.5% glutaraldehyde in 0.1 M Sorenson's buffer (pH 7.4) at 4°C for 4 h. After post‐fixing cell pellets with 1% osmium tetroxide for 2 h at room temperature, the samples were systematically dehydrated. Following complete dehydration and acetone treatment, the specimens were embedded in resin (EMBed 812) overnight. Sections (60–80 nm) were cut using a Leica ultramicrotome. These sections were subsequently mounted on formvar‐coated copper grids and subjected to sequential staining with uranyl acetate and lead citrate. Ultrastructural examination was conducted using a Hitachi TEM.

### 2.12 Assessment of Oxidative Stress

Intracellular ROS levels were assessed using 2′,7′‐dichlorofluorescein diacetate (DCFH‐DA), a cell‐permeable dye that fluoresces upon oxidation by ROS. Mitochondrial ROS were measured using MitoSOX Red Mitochondrial Superoxide Indicator (Thermo Fisher Scientific). Cells were plated in six‐well plates and allowed to adhere overnight. Following experimental treatments, cellular specimens underwent fluorescent probe staining to quantify ROS (DCFH‐DA, 10 μM; MitoSOX Red, 5 μM) (30 min, 37°C) in the dark, followed by rinsing with PBS. ROS production was visualized using fluorescence microscopy, and fluorescence intensity was quantified using ImageJ software (RRID:SCR_003070).

### 2.13 Measurement of Glutathione (GSH) and Glutathione Disulfide (GSSG)

BV‐2 cells were cultured in 96‐well plates under different experimental conditions to measure intracellular levels of GSH and GSSG. After treatment, the GSH and GSSG levels were determined using a luminescence‐based GSH and GSSG assay kit (S0053, Beyotime), following the manufacturer's protocol. The GSH/GSSG ratio was calculated as an indicator of oxidative stress.

### 2.14 Lipid Peroxidation Detection

Lipid peroxidation was measured using the malondialdehyde (MDA) Assay Kit (S0131S, Beyotime), following manufacturer‐provided protocols. Cells were exposed to different experimental compounds for predetermined time intervals. MDA levels were quantified by measuring absorbance at 532 nm using a microplate reader (PerkinElmer). In addition, cells were incubated with 2 μmol/L C11‐BODIPY (Thermo Fisher Scientific) to assess cytosolic lipid peroxidation, and MitoPeDPP (Invitrogen) was used for mitochondrial lipid peroxidation. Fluorescence images were captured using a Nikon Fi3 microscope. Quantitative analysis was performed through ImageJ software (RRID:SCR_003070), with fluorescence intensities normalized to the control group.

### 2.15 Iron Detection

Cells were incubated with 1 μM FerroOrange (Dojindo, Japan) to detect intracellular iron and 5 μM Mito‐FerroGreen (Dojindo, Japan) to detect mitochondrial iron (30 min, 37°C) in a 5% CO_2_ incubator. Fluorescence imaging was conducted via Nikon Fi3 microscope, with subsequent intensity analysis performed.

### 2.16 Measurement of Mitochondrial Membrane Potential (MMP)

MMP evaluation was conducted using tetramethylrhodamine methyl ester (TMRM; Thermo Fisher Scientific). The experimental protocol involved incubating BV2 cells with a 200 nmol/L TMRM (30 min, 37°C) in the dark. Fluorescence images were obtained using a Nikon Fi3 microscope, and fluorescence intensity was quantified with ImageJ software (RRID:SCR_003070), enabling precise quantification of fluorescence intensity across the experimental samples.

### 2.17 Statistical Analysis

All statistical analyses were performed using GraphPad Prism (RRID:SCR_002798). Group comparisons were conducted through independent two‐tailed *t* tests. For multiple group comparisons, one‐way or two‐way ANOVA followed by Tukey's or Sidak's post hoc tests was used as appropriate. All graphical representations were generated using GraphPad Prism (RRID:SCR_002798). Statistical significance was defined as *p* < 0.05.

## 3 Results

### 3.1 SESN2 Expression Is Downregulated in EIU Mouse Retinas

The expression levels of SESN2 in retinal tissue from EIU mice were assessed to investigate its role in inflammation. Samples were collected 24 h post‐operation from all experimental groups. WB analysis and quantitative evaluation revealed a significantly decreased SESN2 expression in the LPS group relative to the controls (Figure S1), suggesting that LPS‐induced inflammation downregulated retinal SESN2 levels. This finding suggests a potential regulatory role for SESN2 in EIU‐associated inflammatory processes.

### 3.2 SESN2 Overexpression Reduces LPS‐Induced Inflammation in EIU

**FIGURE 1:**
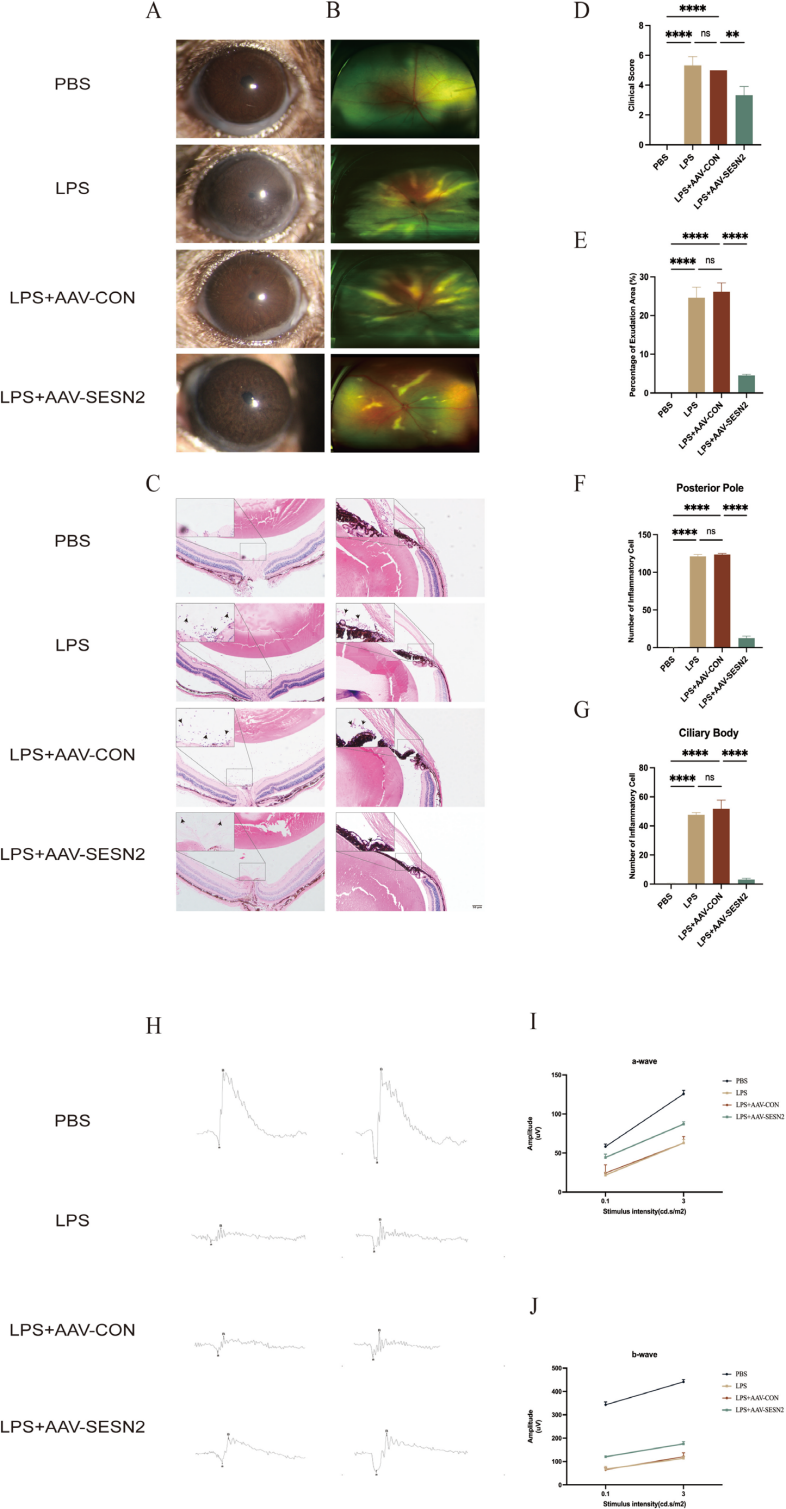
SESN2 alleviates ocular inflammation and improves ERG responses in EIU models. (A) Slit lamp and (B) fundus imaging show suppression of anterior and posterior inflammatory responses in SESN2‐overexpressing mice. (C) H&E‐stained images reveal reduced inflammatory infiltrates in the vitreous cavity with SESN2 overexpression. Statistical analyses indicate significant attenuation of ocular inflammation in SESN2‐overexpressing groups, as evidenced by decreased clinical scores (D), exudation percentage (E), and reduced inflammatory cell numbers in the posterior pole (F) and ciliary body (G). (H) ERG responses were reduced in LPS‐treated groups but were partially recovered with SESN2 overexpression. Quantification of the a‐wave (I) and b‐wave (J) amplitudes. Data are presented as mean ± SEM, *n* = 6 in each group. ****p < 0.0001, **p < 0.01, ns = not significant.

To investigate SESN2's potential anti‐inflammatory mechanisms, AAV vector injections were used to induce SESN2 overexpression, following the protocol outlined in the methods section. Detailed clinical anterior segment examination revealed pronounced inflammatory responses in the LPS and LPS+AAV‐CON groups, characterized by limbal vascular congestion, dilated and tortuous iris vessels, and the presence of hypopyon (Figure 1A). Fundus imaging further revealed significantly decreased retinal exudation in the AAV‐SESN2‐treated group compared to the LPS and LPS+AAV‐CON groups (Figure 1B). Additionally, histological analysis confirmed significant inflammatory cell infiltration in the posterior pole and iris‐ciliary body of the LPS and LPS+AAV‐CON groups (Figure 1C). However, AAV‐mediated SESN2 overexpression notably reduced immune cell accumulation. These results suggest that SESN2 overexpression effectively suppresses intraocular inflammation in the EIU model (Figure 1D–G).

Retinal function was assessed with ERG under scotopic conditions (0.1, and 3 cd s/m^2^). LPS treatment significantly reduced both a‐wave and b‐wave amplitudes (Figure 1H). SESN2 overexpression partially restored these parameters. Quantitative analysis revealed significant inter‐group differences in a‐wave (Figure 1I) and b‐wave (Figure 1J) amplitudes, supporting SESN2's protective role in maintaining retinal function during inflammation.

### 3.3 SESN2 Suppresses Microglial Activation and Attenuates LPS‐Induced Inflammation via Ferroptosis Inhibition

To assess whether SESN2 modulates microglial activation and inflammation in EIU, we examined retinal microglial morphology, inflammatory cytokine expression, and ferroptosis‐associated markers in PBS, LPS, LPS+AAV‐CON, and LPS+AAV‐SESN2 groups.

**FIGURE 2:**
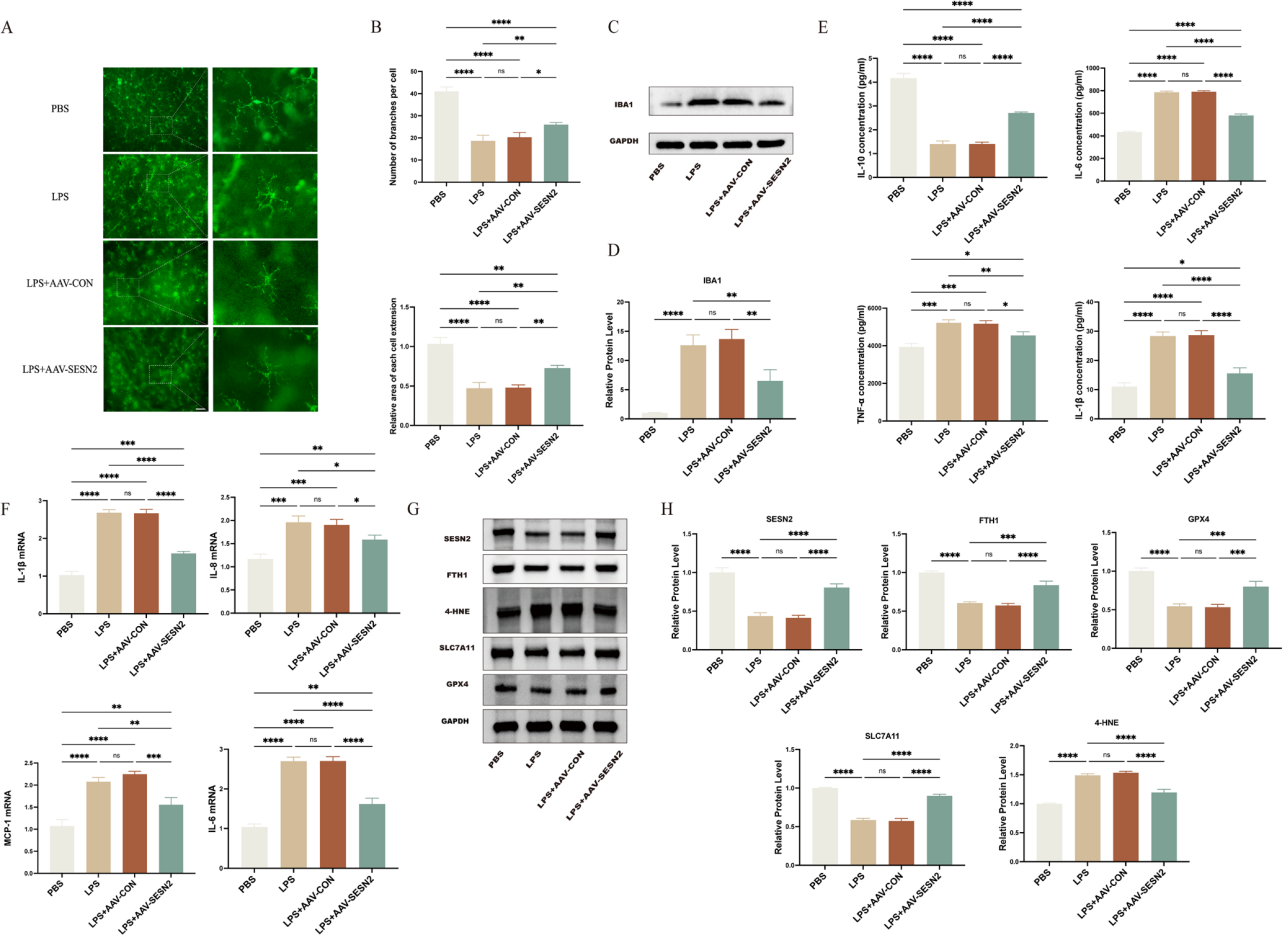
SESN2 modulates microglial morphology, retinal cytokine profiles, and ferroptosis‐associated protein expression in vivo. (A) Retinal flat‐mount immunofluorescence images showing IBA1‐positive microglia. (B) Quantitative analysis of microglial process number and surface coverage. (C, D) Western blot and densitometric analysis of IBA1 protein levels in the retina. (E) Concentrations of IL‐1β, TNF‐α, IL‐6, and IL‐10 in retinal tissue were determined by ELISA. *n* = 3. (F) Relative mRNA expression of the pro‐inflammatory cytokines analyzed by qPCR. *n* = 3. (G) WB analysis of SESN2 and key regulators of ferroptosis (FTH1, GPX4, SLC7A11, and 4‐HNE). *n* = 4. (H) Relative protein expression levels normalized to GAPDH. Data are presented as mean ± SEM. ****p < 0.0001, ***p < 0.001, **p < 0.01, *p < 0.05, ns = not significant. Scale bar: 50 μm.

IBA1 immunofluorescence staining of retinal flat mounts revealed distinct morphological changes in microglia (Figure 2A). In PBS‐treated mice, microglia displayed a ramified morphology with multiple processes and broad coverage, indicative of a resting state. In contrast, LPS exposure induced transformation to an activated morphology with shortened processes. AAV control vector did not alleviate this effect. However, SESN2 overexpression alleviated the LPS‐induced activation of microglia. Quantitative analysis showed significant restoration of microglial branch number and coverage area in the LPS+AAV‐SESN2 group compared to LPS or LPS+AAV‐CON groups (Figure 2B). Western blot analysis of retinal lysates showed that IBA1 protein levels were markedly increased after LPS challenge and partially reduced by AAV‐SESN2 treatment, supporting a modulatory effect on microglial activation (Figure 2C,D). Also, fluorescence images revealed mCherry signal in cells immunopositive for IBA1, suggesting that microglia were among the populations transduced by the AAV vector (Figure S2).

ELISA analysis revealed that LPS significantly increased the levels of pro‐inflammatory cytokines IL‐1β, TNF‐α, and IL‐6, while reducing the anti‐inflammatory cytokine IL‐10. SESN2 overexpression reversed these effects, indicating its role in modulating the inflammatory cytokine balance (Figure 2E). Similarly, qPCR analysis confirmed decreased mRNA levels of pro‐inflammatory cytokines in the LPS+AAV‐SESN2 group compared to the LPS and LPS+AAV‐CON groups (Figure 2F).

Given the crucial role of inflammation in ferroptosis, we examined the relationship between LPS‐induced inflammation and ferroptosis by assessing the expression of key ferroptosis‐related proteins including ferritin heavy chain 1 (FTH1), 4‐hydroxynonenal (4‐HNE), solute carrier family 7 member 11 (SLC7A11), and glutathione peroxidase 4 (GPX4), under various treatment conditions (Figure 2G). While SESN2 expression was downregulated in LPS‐treated groups, SESN2 overexpression significantly reduced 4‐HNE levels, indicating a reduction in lipid peroxidation. FTH1, an iron storage protein, exhibited decreased expression in LPS‐treated groups but was upregulated in the LPS+AAV‐SESN2 group, suggesting that SESN2 is involved in the regulation of iron homeostasis during inflammation. Additionally, SESN2 overexpression led to the upregulation of SLC7A11 and GPX4, key proteins in ferroptosis resistance (Figure 2H). These results indicate that SESN2's protective effects against LPS‐induced inflammation are associated with the mitigation of oxidative damage and the inhibition of ferroptosis.

### 3.4 SESN2 Overexpression Inhibits Ferroptosis In Vitro Through Enhanced Antioxidant Capacity

Building on our previous findings of SESN2's anti‐inflammatory effects in the EIU model, we sought to investigate the underlying molecular mechanisms in LPS‐induced BV2 microglial cells. To assess the influence of SESN2 on pro‐inflammatory cytokine production, oxidative stress, and ferroptosis, we employed both overexpression (via HBLV‐SE) and knockdown (via si‐SE) strategies.

**FIGURE 3:**
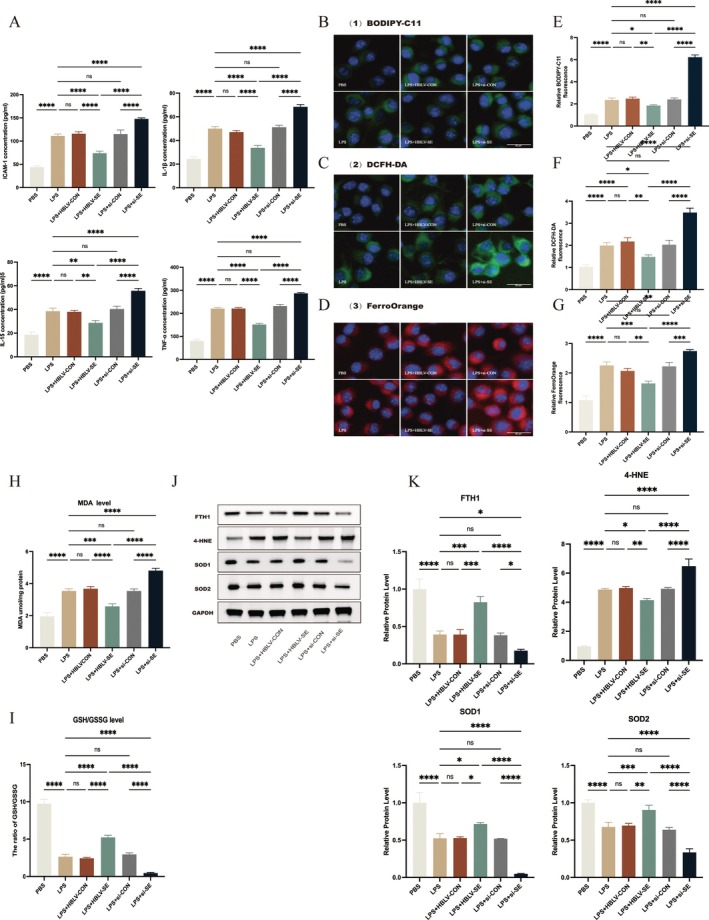
Overexpression of SESN2 suppresses LPS‐induced inflammation and ferroptosis in vitro. (A) Quantification of IL‐1β, TNF‐α, ICAM‐1, and IL‐6 levels by ELISA. *n* = 3. (B) Characterization of lipid ROS in BV2 cells subjected to various treatments, assessed with BODIPY‐C11 staining. (C) Intracellular ROS levels in BV2 cells treated with LPS alone or HBLV, assessed by DCFH‐DA staining. (D) Detection of intracellular Fe^2+^ in BV2 cells under LPS stimulation using FerroOrange. (E–G) Quantitative analysis of fluorescence intensity of BODIPY‐C11, DCFH‐DA, and FerroOrange. *n* = 4. (H) Measurement of MDA content of BV2 cells with or without SESN2 overexpression. (I) GSH/GSSG ratio, reflecting antioxidant capacity. *n* = 3. (J, K) WB and quantitative analysis of ferroptosis‐related proteins. *n* = 4. Data are presented as mean ± SEM. ****p < 0.0001, ***p < 0.001, **p < 0.01, *p < 0.05, ns = not significant.

LPS treatment triggered a significant upregulation of pro‐inflammatory cytokine synthesis, specifically increasing the levels of IL‐6, TNF‐α, IL‐1β, and ICAM‐1 (Figure 3A). Notably, SESN2 overexpression markedly reduced these cytokine levels, while SESN2 knockdown (LPS+si‐SE) further enhanced their expression, indicating SESN2's critical role in the suppression of inflammation. Assessment of oxidative stress and ferroptosis markers revealed that LPS treatment significantly increased lipid peroxidation (BODIPY‐C11), ROS levels (DCFH‐DA assay), and intracellular Fe^2+^ (FerroOrange) compared to controls (Figure 3B–G). SESN2 overexpression attenuated these markers, while SESN2 knockdown exacerbated them, suggesting that SESN2 plays a protective role against oxidative damage and ferroptosis.

Further analysis of MDA, a lipid peroxidation marker, confirmed these observations. LPS treatment significantly increased MDA levels, particularly in the LPS+si‐SE group (Figure 3H), while SESN2 overexpression reduced MDA levels, supporting its role against oxidative damage. The GSH/GSSG ratio, an indicator of antioxidant capacity, was significantly reduced by LPS treatment, reaching its lowest in the LPS+si‐SE group (Figure 3I). SESN2 overexpression restored the GSH/GSSG ratio toward baseline levels, suggesting enhanced antioxidant defense.

Protein expression analysis focusing on oxidative stress and ferroptosis markers indicated that SESN2 overexpression modulated protein expression profiles. Specifically, the WB analysis demonstrated significant upregulation of superoxide dismutase 1 (SOD1), superoxide dismutase 2 (SOD2), and FTH1, concurrent with a significant reduction in 4‐HNE levels (Figure 3J,K). Conversely, SESN2 knockdown resulted in the opposite effect, further confirming its protective role against oxidative stress and ferroptosis.

### 3.5 SESN2 Protects Against Ferroptosis Through Mitochondrial Function Preservation

Considering the central role of mitochondria in oxidative stress, iron regulation, and lipid peroxidation, the impact of SESN2 upregulation on mitochondrial function was further investigated, particularly in the context of ferroptosis protection.

**FIGURE 4:**
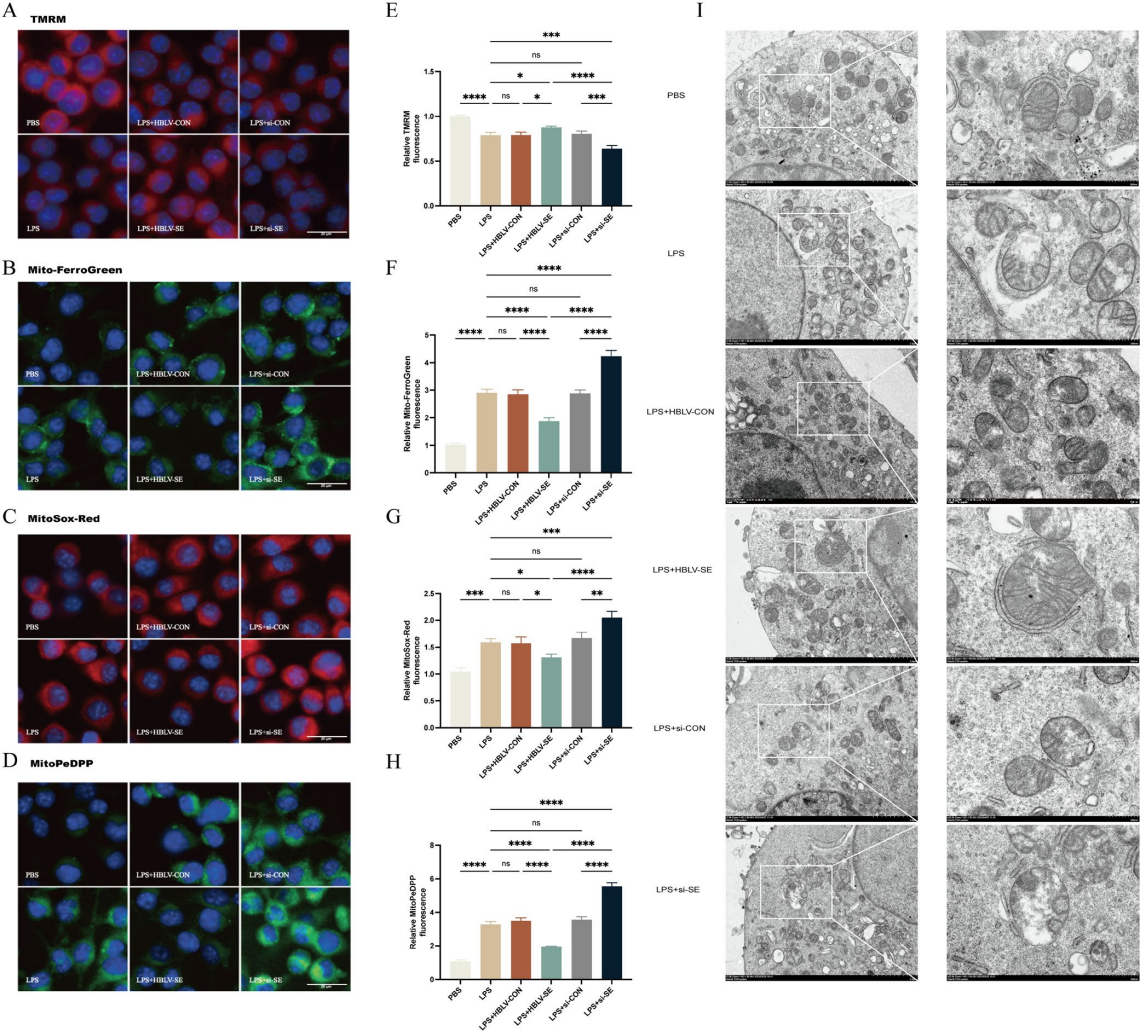
SESN2 protects mitochondrial integrity and reduces oxidative stress in LPS‐induced inflammation. Representative fluorescence images of cells stained with (A) TMRM, (B) Mito‐FerroGreen, (C) MitoSox‐Red, and (D) MitoPeDPP across treatment groups. Quantification of fluorescence intensity for (E) TMRM, (F) Mito‐FerroGreen, (G) MitoSox‐Red, and (H) MitoPeDPP are shown. *n* = 3. (I) TEM images showing mitochondrial integrity across treatment groups. Data are presented as mean ± SEM. ****p < 0.0001, ***p < 0.001, **p < 0.01, *p < 0.05, ns = not significant.

Mitochondrial membrane potential (Δψm), assessed using TMRM, showed a marked reduction in LPS‐treated groups (LPS and LPS+AAV‐CON), indicating compromised mitochondrial function (Figure 4A,E). In contrast, SESN2 overexpression restored Δψm, while SESN2 knockdown further exacerbated the loss of mitochondrial potential, confirming SESN2's role in preserving mitochondrial integrity.

Analysis of mitochondrial‐specific markers revealed that SESN2 overexpression significantly reduced mitochondrial Fe^2+^ accumulation (Mito‐FerroGreen) compared to other LPS‐treated groups (Figure 4B,F). Similarly, mitochondrial ROS production (MitoSox‐Red) increased following LPS exposure but decreased upon SESN2 overexpression (Figure 4C,G). Mitochondrial lipid peroxidation, as assessed by MitoPeDPP, followed a similar trend, with SESN2 overexpression reversing the LPS‐induced increase (Figure 4D,H). Control groups (si‐CON and HBLV‐CON) showed no significant differences, emphasizing SESN2's specific role under inflammatory conditions.

TEM revealed distinct morphological changes in mitochondria across treatment groups (Figure 4I). In the control group, mitochondria exhibited typical morphology, with intact double membranes and well‐defined cristae. LPS treatment caused significant mitochondrial damage, characterized by swelling and disrupted cristae. Notably, SESN2 overexpression preserved mitochondrial structural integrity, while SESN2 knockdown exacerbated structural damage, further confirming the role of SESN2 in maintaining mitochondrial integrity and protecting against LPS‐induced mitochondrial dysfunction.

### 3.6 SESN2 Mediates Ferroptosis Protection Through Nuclear Factor‐Erythroid 2‐Related Factor 2 (Nrf2)‐Dependent Mechanisms

**FIGURE 5:**
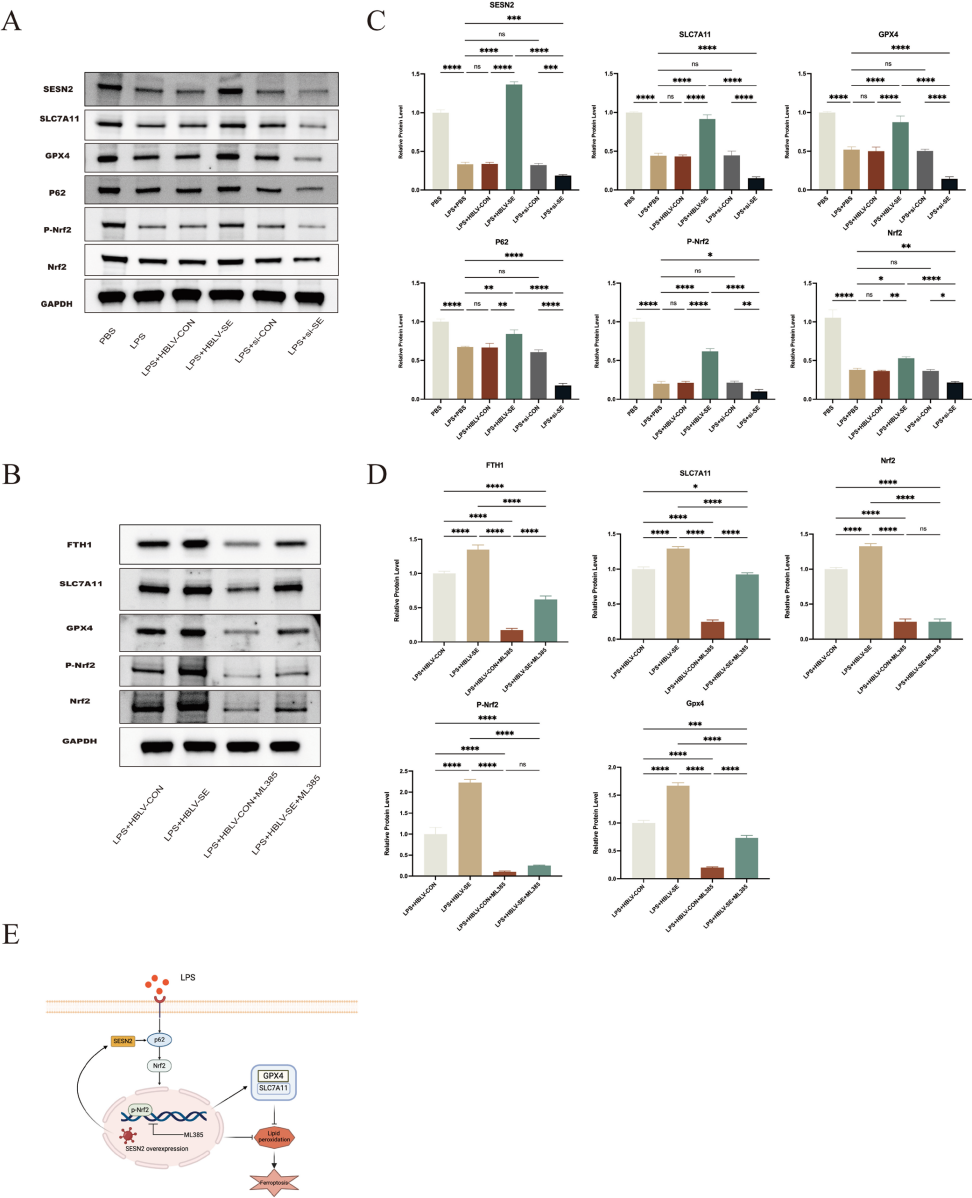
SESN2 regulates ferroptosis through the Nrf2 pathway in LPS‐induced inflammation. (A) WB analysis of SESN2, SLC7A11, GPX4, P62, P‐Nrf2, and Nrf2 expression in vitro across different treatments. (B) WB showing the effect of Nrf2 inhibition using (ML385, 5 μM, 1 μL) on FTH1, SLC7A11, GPX4, P‐Nrf2, and Nrf2 expression across different groups. (C) Quantification of the relative protein levels for SESN2, P62, P‐Nrf2, Nrf2, SLC7A11, and GPX4. (D) Quantification of FTH1, SLC7A11, GPX4, P‐Nrf2, and Nrf2 protein levels in LPS‐induced conditions with and without Nrf2 inhibition. (E) Representation of SESN2‐mediated protection against LPS‐induced ferroptosis via the P62/Nrf2/GPX4 pathway. ****p < 0.0001, ***p < 0.001, **p < 0.01, *p < 0.05, ns = not significant.

Previous research has shown that SESN2 expression interacts with the Nrf2 pathway, contributing to antioxidant activity [33, 34]. To explore this further, our research systematically investigated ferroptosis‐associated protein expressions in LPS‐treated BV2 microglial cells. The results demonstrated that SESN2 overexpression significantly increased the expression of Nrf2, phosphorylated Nrf2 (P‐Nrf2), and ferroptosis resistance markers SLC7A11 and GPX4 (Figure 5A,C). In contrast, SESN2 knockdown reduced the expression of these proteins, suggesting that SESN2 confers protection against ferroptosis by activating the Nrf2 pathway, which in turn upregulates key ferroptosis‐inhibitory proteins. Elevated p62 expression in the SESN2 overexpression group further supported p62's involvement in ferroptosis resistance through the Nrf2 pathway.

To confirm the role of Nrf2 in the protective effects of SESN2, BV2 cells were co‐treated with the Nrf2 inhibitor ML385. SESN2 overexpression significantly increased levels of Nrf2, P‐Nrf2, SLC7A11, GPX4, and FTH1 compared to LPS‐treated controls (Figure 5B,D). However, co‐treatment with ML385 resulted in a significant suppression of protein expression, confirming that SESN2's protective effects against ferroptosis are primarily mediated through the Nrf2 pathway. Collectively, these findings indicate that SESN2 reduces LPS‐induced ferroptosis via the p62/Nrf2/GPX4 signaling axis (Figure 5E).

## 4 Discussion

This comprehensive investigation examined the protective mechanisms of SESN2 against LPS‐induced inflammatory and ferroptotic processes across experimental model systems. Our findings demonstrate that SESN2 overexpression mitigates LPS‐induced oxidative stress, preserves mitochondrial function, and inhibits ferroptosis through activation of the p62/Nrf2/SLC7A11/GPX4 molecular signaling cascade. These results offer new insights into acute uveitis mechanisms and propose SESN2 as a potential strategic intervention for inflammation‐related ocular pathologies.

Previous studies have highlighted the critical role of ferroptosis in inflammatory conditions and neurodegenerative diseases [35, 36, 37, 38]. Ferroptosis is characterized by iron‐dependent lipid peroxidation leading to cell death, and it has been implicated in various retinal disorders, including uveitis [17, 39, 40]. Our study extends the current literature by demonstrating that LPS induces ferroptosis in retinal tissues and microglial cells, as evidenced by increased lipid peroxidation, iron accumulation, and downregulation of ferroptosis‐related proteins such as GPX4 and SLC7A11.

The function of SESN2 in controlling inflammation is well‐documented, particularly in reducing pro‐inflammatory cytokine release and regulating autophagy [41, 42, 43]. Studies have shown that SESN2 modulates the NF‐κB signaling cascade, resulting in reduced secretion of pro‐inflammatory mediators, while also regulating autophagy and metabolism to alleviate inflammation [44]. In our experiments, SESN2 expression was downregulated in the retinas of EIU mice, suggesting that inflammation suppresses SESN2 levels. Overexpression of SESN2 via AAV vectors restored its levels and led to a significant reduction in inflammatory signs, both clinically and histologically. This was accompanied by a decrease in pro‐inflammatory cytokines such as TNF‐α, IL‐6, and IL‐1β in retinal tissues. Furthermore, its overexpression preserved retinal function, as indicated by restored a‐ and b‐wave amplitudes in ERG recordings.

Beyond its anti‐inflammatory properties, SESN2 plays a key role in regulating oxidative stress [45, 46]. LPS‐induced inflammation typically triggers excessive ROS generation, leading to cellular damage [47, 48]. Our data demonstrated that SESN2 overexpression significantly reduced ROS levels and elevated antioxidant enzymes expression (SOD1, SOD2) in BV2 microglial cells exposed to LPS. Furthermore, SESN2 enhanced cellular antioxidant capacity by activating the Nrf2 signaling pathway, reducing lipid peroxidation as indicated by lower 4‐HNE levels, and restoring the GSH/GSSG ratio, highlighting SESN2's central role in oxidative stress mitigation.

A notable finding in our study is the proposed involvement of the p62/Nrf2/GPX4 axis acting downstream of SESN2 in ocular inflammation. Nrf2, a key transcription factor in cellular antioxidant activities, regulates the expression of critical ferroptosis inhibitory genes, including SLC7A11 and GPX4 [49, 50]. It promotes p62‐mediated selective autophagic degradation of Keap1, relieving Keap1‐dependent ubiquitination and degradation of Nrf2. This stabilizes Nrf2 and facilitates its nuclear translocation to activate antioxidant gene expression. Our data demonstrate that SESN2 overexpression leads to increased Nrf2 phosphorylation and activation, which upregulates SLC7A11 and GPX4, essential regulators of the glutathione antioxidant system and inhibitors of ferroptosis. Additionally, SESN2 enhances PINK1/Parkin‐dependent mitophagy to reduce mitochondrial ROS, indirectly supporting Nrf2 signaling [51, 52]. Inhibition of Nrf2 using ML385 attenuated SESN2‐mediated upregulation of SLC7A11 and GPX4, leading to increased oxidative stress and ferroptosis markers, thus confirming the central role of Nrf2 in SESN2‐mediated protection. Evidence for the p62/Nrf2/GPX4 pathway has been reported in diverse inflammatory diseases, including intestinal ischemia–reperfusion injury, diabetic retinopathy, and liver injury, where ferroptosis is implicated in pathogenesis via oxidative lipid damage and inflammation [53, 54, 55]. In the eye, Nrf2 deficiency aggravates EIU and light‐induced retinal degeneration, underscoring the relevance of this axis to retinal homeostasis [56, 57]. Although our data demonstrate that SESN2 overexpression activates Nrf2 and upregulates SLC7A11 and GPX4, we did not directly interrogate p62's contribution—for example, through p62 knockdown or point‐mutant rescue. Therefore, while the p62/Nrf2/GPX4 model is mechanistically plausible and supported by extensive literature, we conservatively frame it here as a working hypothesis and highlight it as a priority for future experimental validation in ocular inflammation models.

Mitochondrial dysfunction is a hallmark of ferroptosis and contributes to the amplification of oxidative stress [58]. Our study showed that SESN2 overexpression preserved mitochondrial membrane potential, reduced mitochondrial iron and ROS accumulation, and maintained mitochondrial integrity in LPS‐treated BV2 cells. TEM images revealed that SESN2 prevented the morphological changes associated with ferroptosis, such as mitochondrial shrinkage and cristae disruption. These findings indicate that SESN2 not only acts through antioxidant pathways but also directly influences mitochondrial health to exert its protective effects.

While our study provides significant insights, there are limitations that require discussion. First, although this established scoring system served as a practical tool to evaluate ocular inflammation anteriorly, it presents certain defects and lacks complete objectivity. The grading of clinical criteria inevitably introduces subjective interpretation that may compromise the precision, and potentially lead to inter‐observer variability. While semi‐quantitative scoring was adequate for the purposes of our current validation, its limitations highlight the need to consider adopting quantitative approaches such as laser flare photometry and anterior segment optical coherence tomography in future investigations. Additionally, the upstream regulatory mechanisms of SESN2 are not fully understood; elucidating the signaling pathways that regulate SESN2 expression could reveal additional therapeutic targets. Second, although we demonstrated the involvement of the P62/Nrf2 pathway, other pathways such as AMP‐activated protein kinase (AMPK) and mammalian target of rapamycin (mTOR) may also interact with SESN2 and contribute to its effects [51, 59]. Future studies should explore these interactions to fully elucidate the network of signaling pathways involved. Third, our study focused on acute inflammation models. Chronic uveitis involves different pathogenic mechanisms, and the role of SESN2 in chronic inflammation and its potential long‐term therapeutic effects needs further investigation. Moreover, while BV2 cells are a widely used model for microglia, they are immortalized cells that may not fully replicate the behavior of primary microglia. Confirming our findings in primary microglial cultures or in vivo microglial studies would strengthen the translational relevance of our results. Finally, an important limitation is the lack of definitive evidence regarding the cell type–specificity of AAV‐SESN2 in the inflamed retina. Although our co‐immunostaining analyses revealed partial overlap between mCherry expression and IBA1‐positive microglia, these data alone cannot exclude the possibility that other resident or infiltrating cells (e.g., Müller cells, retinal ganglion cells, vascular endothelial cells, or iris stromal cells) may also be transduced, particularly under inflammatory conditions where AAV tropism may broaden. Thus, the protective effects of SESN2 may, at least in part, involve actions in additional cell populations. Future work employing single‐cell RNA sequencing, cell type–specific promoters, Cre‐Lox systems, or other lineage‐tracing strategies will be essential to precisely define the cellular targets and to determine whether the observed anti‐inflammatory effects arise predominantly from microglial modulation or also engage other immune compartments.

In conclusion, this study highlights that SESN2 exerts protective effects in LPS‐induced ocular inflammation through P62/Nrf2/GPX4 signaling pathway‐mediated regulation of ferroptosis. These findings provide insights for future therapeutic strategies targeting SESN2, with potential clinical applications in acute uveitis and other neuroinflammatory and retinal diseases associated with acute inflammation.

## Author Contributions

X.Z. and J.Y. wrote the main manuscript text. Y.Y. and W.Z. performed the methodology and software analysis. H.X. and E.C. contributed to visualization and validation. Z.L., H.L., and M.L. were involved in the investigation. Y.W. provided resources for the study. X.Z. handled data curation. S.G. and L.H. were responsible for data curation and validation. J.P. contributed to writing – review and editing, resources, and methodology. H.F. handled project administration, methodology, and conceptualization. P.Z. supervised the study, contributed to writing – review and editing, and managed project administration, investigation, and funding acquisition. All authors read and approved the final manuscript.

## Ethics Statement

All protocols for animal studies were reviewed and approved by the Institutional Animal Care and Use Committee of Xinhua Hospital (Approval No: XHEC‐NSFC‐2023‐145).

## Conflicts of Interest

The authors declare no conflicts of interest.

## Supporting information


**Figure S1:** SESN2 expression is downregulated in EIU mouse retinas. (A) WB analysis showing differential SESN2 expression in EIU models. (B) Quantitative analysis of SESN2 expression levels.

**Figure S2:** Validation of AAV‐Sesn2 transduction efficiency and cellular specificity in the retina. (A) Retinal flat‐mount immunofluorescence images showing mCherry and IBA1 staining in PBS, LPS, LPS+AAV‐CON, and LPS+AAV‐SESN2 groups. (B) Quantification of mCherry+/IBA1+ double‐positive cells across groups.

**Table S1:** Primer sequences.
**Table S2:** EIU clinical scoring criteria (slit lamp).

## Data Availability

The data that support the findings of this study are available in the Materials and Methods, Results, and/or Supporting Information of this article.
