# Supplementary figures and images for: Sestrin2 Exerts a Novel Protective Effect Against LPS‐Induced Ferroptosis via the Nrf2–SLC7A11–GPX4 Signaling Axis
Source: FASEB J. 2025 Nov 26;39(22):e71251. doi: 10.1096/fj.202501348RRR (PMC12651112; doi:10.1096/fj.202501348RRR)

A

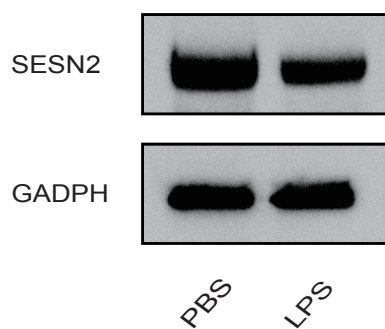

B

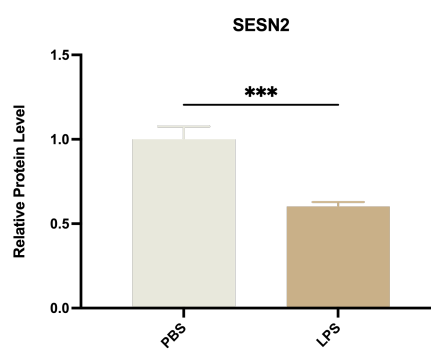

Supplement: Supplementary file 1 — Figure S1: SESN2 expression is downregulated in EIU mouse retinas. (A) WB analysis showing differential SESN2 expression in EIU models. (B) Quantitative analysis of SESN2 expression levels. [file FSB2-39-e71251-s001.pdf]

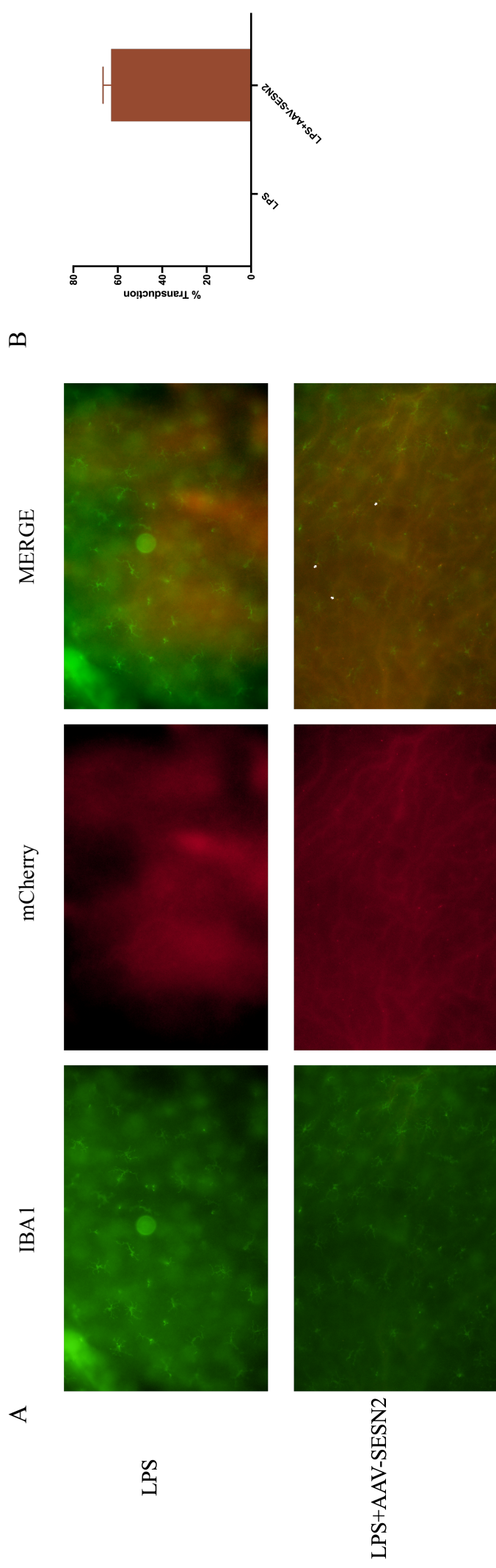

Supplement: Supplementary file 2 — Figure S2: Validation of AAV‐Sesn2 transduction efficiency and cellular specificity in the retina. (A) Retinal flat‐mount immunofluorescence images showing mCherry and IBA1 staining in PBS, LPS, LPS+AAV‐CON, and LPS+AAV‐SESN2 groups. (B) Quantification of mCherry+/IBA1+ double‐positive cells across groups. [file FSB2-39-e71251-s003.pdf]
