# Supplementary material for: Sestrin2 Exerts a Novel Protective Effect Against LPS‐Induced Ferroptosis via the Nrf2–SLC7A11–GPX4 Signaling Axis
Source: FASEB J. 2025 Nov 26;39(22):e71251. doi: 10.1096/fj.202501348RRR (PMC12651112; doi:10.1096/fj.202501348RRR)
Supplement: Supplementary file 3 — Table S1: Primer sequences. Table S2: EIU clinical scoring criteria (slit lamp). [file FSB2-39-e71251-s002.docx]

Table S1. Primer sequences.

| Indicators | Forward (5’-3’) | Reverse (5’-3’) |
| --- | --- | --- |
| IL-1β | GCAACTGTTCCTGAACTCAACT | ATCTTTTGGGGTCCGTCAACT |
| IL-6 | CCAAGAGGTGAGTGCTTCCC | CTGTTGTTCAGACTCTCTCCCT |
| IL-8 | CAAGGCTGGTCCATGCTCC | TGCTATCACTTCCTTTCTGTTGC |
| MCP-1 | TTAAAAACCTGGATCGGAACCAA | GCATTAGCTTCAGATTTACGGGT |

Table S2. EIU Clinical Scoring Criteria (Slit Lamp)

| Clinical signs | Grade of Signs | Score |
| --- | --- | --- |
| Iris blood vessel dilation | Absent | 0 |
|  | Mild | 1 |
|  | Moderate | 2 |
|  | Severe | 3 |
| Fibrin exudation | Absent | 0 |
|  | Minor | 1 |
|  | Significant | 2 |
| Hypopyon | Absent | 0 |
|  | Present | 1 |
| Pupil | Normal | 0 |
|  | Synechia | 1 |
| Maximum possible score |  | ­7 |

Table S2. Clinical evaluation criteria (slit lamp). The table shows the grading and scoring of clinical signs in endotoxin-induced uveitis (EIU), including iris blood vessel dilation, fibrin exudation, hypopyon, and pupil status. The maximum possible score is 7.
